# 52例原发中枢神经系统淋巴瘤患者临床特征及预后分析

**DOI:** 10.3760/cma.j.cn121090-20241130-00515

**Published:** 2025-08

**Authors:** 芳 包, 森 李, 钊 刘, 红梅 景

**Affiliations:** 1 北京大学第三医院血液内科，北京 100191 Department of Hematology, Peking University Third Hospital, Beijing 100191, China; 2 首都医科大学三博脑科医院功能神经外科，北京 100093 Department of Functional Neurosurgery, Sanbo Brain Hospital, Capital Medical University, Beijing 100093, China

**Keywords:** 淋巴瘤, 中枢神经系统, 预后, 自体造血干细胞移植, 嵌合抗原受体T细胞免疫治疗, Lymphoma, Central nervous system, Prognosis, Autologous hematopoietic stem cell transplantation, Chimeric antigen receptor T-cell immunotherapy

## Abstract

**目的:**

探讨原发中枢神经系统淋巴瘤（PCNSL）的临床特征、治疗方案以及预后相关因素。

**方法:**

纳入2013年1月至2023年12月北京大学第三医院收治的52例PCNSL患者临床资料，对患者临床特征、治疗方案以及预后相关因素进行回顾性分析。通过单因素及多因素Cox比例风险模型分析PCNSL患者无进展生存（PFS）和总生存（OS）的影响因素。

**结果:**

所有患者中位发病年龄为57（23～87）岁，男女比例为1.08∶1。神经功能障碍（71.2％）和颅内高压（57.7％）为常见的临床表现。肿瘤累及深部脑组织，呈多灶病变。49例可评估疗效，中位随访时间为23（95％ *CI*：8.6～37.4）个月，2年、5年PFS率分别为56.4％（95％*CI*：42.2％～68.3％）、36.3％（95％*CI*：17.3％～53.4％），总生存（OS）率分别为75.5％（95％*CI*：61.7％～87.2％）、66.0％（95％*CI*：43.9％～78.3％）。单因素Cox回归分析显示，年龄>60岁（*HR*＝3.436，95％ *CI*：1.008～11.710，*P*＝0.049）、美国纪念斯隆-凯特琳癌症中心（MSKCC）评分系统分层高危（*HR*＝22.130，95％ *CI*：4.736～103.400，*P*<0.001）为OS的不良预后因素，接受auto-HSCT治疗（*HR*＝0.223，95％ *CI*：0.077～0.643，*P*＝0.006）可延长OS期。多因素Cox回归分析结果显示，接受含大剂量甲氨蝶呤（HD-MTX）化疗（*HR*＝0.082，95％ *CI*：0.008～0.873，*P*＝0.038）、接受auto-HSCT治疗（*HR*＝0.151，95％ *CI*：0.030～0.747，*P*＝0.020）是OS的独立预后因素。

**结论:**

PCNSL患者中，高龄及预后危险分层高危患者预后较差。采用含HD-MTX治疗方案以及进行auto-HSCT可改善患者生存。

## Abstract (en)

**Objective:**

To investigate the clinical characteristics, treatment and prognostic factors in patients with primary central nervous system lymphoma（PCNSL）.

**Methods:**

This retrospective study included 52 patients with PCNSL treated in Peking University Third Hospital between January 2013 and December 2023. An analysis was conducted on the clinical characteristics, treatment and prognostic factors of the patients. Univariate and multivariate Cox proportional hazards models were used to determine factors associated with progression-free survival and overall survival（OS）.

**Results:**

In the overall cohort, the median age at diagnosis was 57（range, 23–87）years, with a male/female ratio of 1.08:1. Neurological dysfunction（71.2％）and intracranial hypertension（57.7％）were common clinical manifestations. The tumors involved the deep brain tissue and presented as multifocal lesions. Treatment efficacy was evaluable in 49 patients, with a median follow-up of 23（95％ *CI*: 8.6–37.4）months. The 2- and 5-year PFS rates were 56.4％（95％ *CI*: 42.2％–68.3％）and 36.3％（95％ *CI*: 17.3％–53.4％）, respectively, whereas the OS rates were 75.5％（95％ *CI*: 61.7％–87.2％）and 66.0％（95％ *CI*: 43.9％–78.3％）, respectively. By univariate Cox regression analysis, age >60 years（*HR*＝3.436, 95％ *CI*: 1.008–11.710, *P*＝0.049）and Memorial Sloan-Kettering Cancer Center grade 3 tumor（*HR*＝22.10, 95％ *CI*: 4.736–103.400, *P*<0.001）were prognostic factors for worse OS, whereas auto-hematopoietic stem cell transplantation（HSCT）as consolidation therapy was significantly associated with longer OS（*HR*＝0.223, 95％*CI*: 0.077–0.643, *P*＝0.006）. By multivariate Cox regression analysis, high-dose methotrexate chemotherapy（*HR*＝0.082, 95％*CI*: 0.008–0.873, *P*＝0.038）and auto-HSCT（*HR*＝0.151, 95％*CI*: 0.030–0.747, *P*＝0.020）were independent predictors of prolonged OS.

**Conclusion:**

The prognosis was poor in patients with PCNSL who are elderly and those at high risk according to risk stratification. Adopting treatment regimens containing high-dose methotrexate and performing auto-HSCT can improve survival in patients with PCNSL.

原发中枢神经系统淋巴瘤（PCNSL）是一类罕见的结外非霍奇金淋巴瘤，病灶仅局限累及脑实质、脊髓、软脑膜和眼，而无全身性淋巴瘤受累的证据。PCNSL发病率占非霍奇金淋巴瘤的1％～2％、占结外淋巴瘤的4％～6％、占原发脑肿瘤的4％，年发病率为（0.4～0.5）/10万[1]–[3]。>60岁患者发病率有上升趋势[4]。由于PCNSL临床罕见，目前尚无统一标准的一线治疗方案。PCNSL预后差，5年总生存（OS）率仅为30.5％[5]–[6]。PCNSL因特殊的发病位置、特异的分子遗传学异常及生物学特点，被世界卫生组织（WHO）定义为淋巴瘤的一个独特亚型[7]。第5版WHO造血与淋巴组织肿瘤分类[8]将PCNSL归类于原发性免疫豁免部位淋巴瘤。本研究总结既往临床经验，为开拓新的诊疗规范提供有益数据。

## 病例与方法

1. 临床资料：回顾性分析2013年1月至2023年12月北京大学第三医院收治的52例PCNSL患者的临床资料。患者经开颅手术切除或立体定向穿刺活组织检查获得的组织，经病理学诊断确诊为弥漫大B细胞淋巴瘤（DLBCL）。DLBCL病理亚型分型依据Hans模型分为生发中心B细胞（GCB）亚型和非生发中心B细胞（non-GCB）亚型，部分患者送检二代测序基因检测。双打击指免疫荧光染色或标准细胞遗传学检测伴有MYC和BCL2基因重排，双表达指没有相应的基因重排，但免疫组化染色有MYC和BCL2蛋白高表达（MYC≥40％和BCL2≥50％）。同时收集PET-CT及骨髓穿刺等相关辅助检查资料，明确患者无中枢神经系统以外受累的证据，剔除以中枢神经系统复发为表现的全身性非霍奇金淋巴瘤患者，符合WHO 2016年版PCNSL诊断[9]。对于仅有脑脊膜或神经根受累而无脑实质占位的患者，其淋巴瘤诊断可由脑脊液细胞学和流式细胞术检查证实。眼内淋巴瘤通过前房穿刺，或诊断性玻璃体切除获取组织病理学结果，诊断符合《玻璃体视网膜淋巴瘤诊断及推荐治疗中国专家共识（2024年版）》[10]诊断标准。本研究符合2024年修订的《赫尔辛基宣言》的要求，患者及家属均知情同意。本研究经北京大学第三医院医学科学研究伦理委员会批准，批件号为（2022）医伦审第（162-02）号。

2. 预后评分：预后评分采用国际结外淋巴瘤协作组（IELSG）[11]和美国纪念斯隆-凯特琳癌症中心（MSKCC）预后评分系统[12]。IELSG评分系统包括年龄（≤60岁为0分，>60岁为1分）、美国东部肿瘤协作组（ECOG）体能状态评分（<2分为0分，≥2分为1分）、LDH水平（正常为0分，升高为1分）、是否为颅内深部组织受累（包括侧脑室旁、基底节、脑干、小脑等；未受累为0分，受累为1分）；低危组为0～1分，中危组为2～3分，高危组为4～5分。MSKCC评分系统包括年龄和Karnofsky功能状态（KPS）评分：低危组为年龄<50岁，中危组为年龄≥50岁且KPS评分≥70分，高危组为年龄≥50岁且KPS评分<50分。

3. 疗效评价：采用国际PCNSL协作组（IPCG）制定的PCNSL疗效评价标准[13]进行疗效评估，包括完全缓解（CR）、未确认完全缓解（CRu）、部分缓解（PR）、疾病稳定（SD）、疾病进展（PD）。客观缓解率（ORR）：基于RECIST评价标准，定义为肿瘤体积缩小达到预先规定值并能维持最低时限要求的患者比例，即CR、CRu和PR的比例之和。

4. 随访：采用查阅门诊、住院病例及电话方式进行随访，随访截止时间为2024年6月6日。无进展生存（PFS）期定义为PCNSL确诊至疾病进展、复发、死亡或随访截止的时间。OS期定义为PCNSL确诊至死亡或随访截止的时间。

5. 统计学处理：计量资料采用Kolmogorov-Smirnov进行正态性检验，符合正态分布的计量资料采用“*M*（95％置信区间）”进行描述，采用独立样本*t*检验进行统计学分析。不符合正态分布的计量资料，采用“*M*（范围）”进行描述，采用非参数检验进行组间差异的统计学分析。计数资料采用“例数（％）”进行描述，组间差异采用卡方检验进行统计学分析。生存分析采用单因素和多因素Cox比例风险回归评价分类变量组与PFS、OS之间的关系。采用SPSS 29.0和Graphpad prism 10.2软件进行数据分析。所有检验以双侧*P*<0.05为差异具有统计学意义。

## 结果

1. 临床特征：共有52例PCNSL患者纳入研究。如表1所示，男27例、女25例；中位发病年龄57（23～87）岁，其中>60岁患者17例（32.7％）。患者初诊时最常见的临床表现为神经功能障碍（肢体感觉或运动障碍、言语不利、视野缺损或颅神经损害，占71.2％），其次为颅内高压症状（头痛、恶心、呕吐、视乳头水肿等，占57.7％）、神经精神症状（认知障碍如记忆力障碍、定向力障碍、计算力障碍、人格障碍、意识障碍，占25.0％）和癫痫（7.7％）。诊断时51.9％的患者呈多部位受累；71.2％的患者病变位于脑组织深部（包括侧脑室旁、基底节、胼胝体、脑干、小脑等）。最常见受累部位为额叶（34.6％）和基底节（30.8％），其次为顶叶（17.3％）和胼胝体（17.3％）（图1）。24例（46.2％）患者通过手术切除方式获取病理组织标本，余28例（53.8％）患者通过穿刺获取。52例患者病理组织学均为DLBCL。根据Hans分类，41例（83.7％）为non-GCB亚型。46例患者行免疫组化法检测Bcl-2和c-Myc，双表达阳性患者为16例（34.8％）。16例患者进行MYC基因FISH检测，无双打击患者。仅20例患者初治前进行脑脊液检测，其中10例（50％）存在脑脊液蛋白含量升高。

**表1:** 52例原发中枢神经系统淋巴瘤患者的临床特征

临床特征	统计值
年龄［岁，*M*（范围）］	57（23～87）
性别（例，男/女）	27/25
原发部位［例（％）］	
脑	47（90.4）
眼	4（7.7）
脊髓	1（1.9）
首发症状［例（％）］	
神经功能障碍	37（71.2）
颅内高压症状	30（57.7）
神经精神症状	13（25.0）
癫痫	4（7.7）
病灶数量［例（％）］	
单病灶	25（48.1）
多病灶	27（51.9）
深部脑组织受累［例（％）］	37（71.2）
血清LDH升高［例（％）］	10（19.2）
ECOG体能状态评分≥2分［例（％）］	18（34.6）
脑脊液蛋白升高［例（％）］（20例）	10（50.0）
病理类型［例（％）］（49例）	
GCB型	8（16.3）
non-GCB型	41（83.7）
双表达阳性［例（％）］（46例）	16（34.8）
Ki-67阳性指数［例（％）］（48例）	
≥80％	33（68.8）
<80％	15（31.2）
IELSG预后危险分层［例（％）］（20例）	
低危	7（35.0）
中危	10（50.0）
高危	3（15.0）
MSKCC评分系统［例（％）］	
低危	14（26.9）
中危	27（51.9）
高危	11（21.2）
基因分型［例（％）］（15例）	
MCD样亚型	7（46.7）
TP53突变亚型	4（26.7）
BN2样亚型	1（6.7）
其他亚型	3（20.0）

**注** LDH：乳酸脱氢酶；ECOG：美国东部肿瘤协作组；GCB：生发中心B细胞；non-GCB：非生发中心B细胞；IELSG：国际结外淋巴瘤协作组；MSKCC：美国纪念斯隆-凯特琳癌症中心

**图1:**
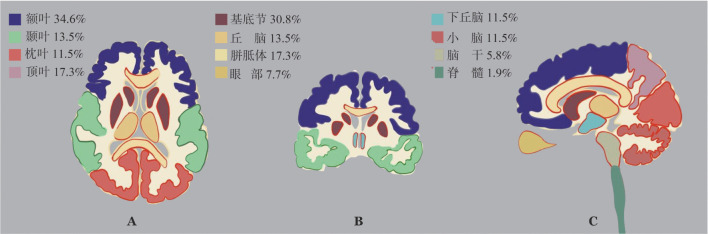
52例原发中枢神经系统淋巴瘤患者的受累部位分布 **A** 轴位图；**B** 冠状位图；**C** 矢状位图

15例患者送检肿瘤组织行二代测序检测。共检测到67个不同的基因突变。基因突变中以MYD88（11例，73.3％）、PIM1（11例，73.3％）最常见，DTX1次之（6例，40.0％），其余较常见的依次为BTG2（5例，33.3％）、CD79B（4例，26.7％）、ETV6（4例，26.7％）、CARD11（4例，26.7％）、IRF4（4例，26.7％）、TP53（4例，26.7％）。参照LymphPlex算法[14]，MCD样亚型最常见，占46.7％，其次为TP53突变亚型（26.7％）、其他亚型（20.0％）、BN2样亚型（6.7％）。

2. 一线诱导及巩固维持治疗：52例PCNSL患者中，2例拒绝治疗，1例在接受1个疗程化疗后失访，49例可评估疗效。一线诱导治疗：42例接受含高剂量甲氨蝶呤（HD-MTX）化疗，其中17例联合布鲁顿酪氨酸激酶抑制剂（BTKi），2例联合来那度胺，1例联合BTKi及来那度胺；2例无HD-MTX化疗患者分别接受了R-EPOCH（利妥昔单抗+依托泊苷+泼尼松+长春新碱+环磷酰胺+蒽环类药物）联合替莫唑胺方案和R-CHOPE（利妥昔单抗+环磷酰胺+蒽环类药物+长春地辛+泼尼松+依托泊苷）方案诱导治疗；5例患者采用无化疗药物治疗方案（仅含靶向药物及新药），其中4例为抗CD20单抗、BTKi（奥布替尼或伊布替尼）联合免疫调节剂（IMiD，泊马度胺或来那度胺），1例为抗CD20单抗联合来那度胺。

49例患者总CR率为69.4％、PR率为16.3％、ORR为85.7％。接受含HD-MTX化疗患者的CR率为71.4％、PR率为16.7％、ORR为88.1％。亚组分析中，HD-MTX同时联合新药的患者的CR率和ORR略高于未联合新药的患者，CR率分别为75.0％和68.2％、ORR分别为90.0％和86.4％，差异均无统计学意义（均*P*>0.05）。无HD-MTX化疗方案的2例患者均经一线诱导治疗达到CR。5例接受无化疗药物方案治疗的患者，2例达CR、1例PR、2例PD（表2）。

**表2:** 49例可评估疗效的原发中枢神经系统淋巴瘤患者的一线诱导治疗方案及疗效［例（％）］

疗效评估	含HD-MTX化疗（42例）		无HD-MTX化疗方案（2例）	无化疗药物方案（5例）
	联合新药（20例）	未联合新药（22例）		
CR	15（75.0）	15（68.2）	2（100）	2（40.0）
PR	3（15.0）	4（18.2）	0（0）	1（20.0）
SD	1（5.0）	0（0）	0（0）	0（0）
PD	1（5.0）	3（13.6）	0（0）	2（40.0）

**注** HD-MTX：高剂量甲氨蝶呤；CR：完全缓解；PR：部分缓解；SD：疾病稳定；PD：疾病进展

经过一线诱导治疗后，34例患者达到CR，并进入巩固维持治疗阶段，其中12例接受auto-HSCT巩固治疗，16例接受药物巩固维持治疗，1例接受放疗，5例未接受任何形式巩固维持治疗。12例接受auto-HSCT的患者中，6例在auto-HSCT后加用BTKi维持治疗。16例接受药物巩固维持治疗患者中，9例采用BTKi单药维持，3例采用含来那度胺方案（1例为单药，2例为联合治疗，分别联合替莫唑胺、MTX）维持，4例采用含HD-MTX方案（1例为单药，3例为联合治疗，联合药物包括利妥昔单抗、替莫唑胺、来那度胺）巩固，3例采用含替莫唑胺方案（均为联合治疗，联合药物包括MTX、来那度胺、利妥昔单抗）巩固维持。

12例接受一线auto-HSCT巩固治疗患者中，后续BTKi维持治疗的6例患者截至末次随访均无复发，未接受后续BTKi维持治疗的6例患者中，1例auto-HSCT后6个月PD。16例接受药物治疗的患者，8例PD。28例接受巩固维持治疗的患者，共9例PD（32.1％）。5例未接受任何巩固维持治疗的患者，3例PD（60.0％）。

3. 预后分析：中位随访23（95％ *CI*：8.6～37.4）个月，在49例可随访患者中，24例（49.0％）出现疾病复发或进展，中位治疗周期为6（1～9）周期，中位PFS期为31（95％*CI*：13～96）个月，2年、5年PFS率分别为56.4％（95％*CI*：42.2％～68.3％）、36.3％（95％*CI*：17.3％～53.4％）（图2A）。49例患者中，14例死亡，其中10例死于PD，4例死于诱导化疗期间严重感染。中位OS期为79（95％*CI*：35.7～152.0）个月。2年、5年OS率分别为75.5％（95％*CI*：61.7％～87.2％）、66.0％（95％*CI*：43.9％～78.3％）（图2B）。

**图2:**
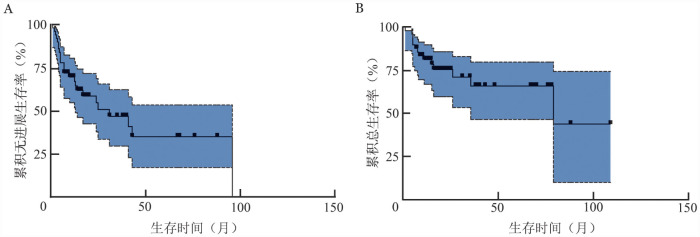
49例原发中枢神经系统淋巴瘤患者的无进展生存曲线（A）和总生存曲线（B）

Kaplan-Meier单因素生存分析显示：年龄> 60岁（*HR*＝3.436，95％*CI*：1.008～11.710，*P*＝0.049）、MSKCC评分系统分层高危（*HR*＝22.130，95％*CI*：4.736～103.400，*P*<0.001）为OS的不良预后因素，接受auto-HSCT治疗（*HR*＝0.223，95％*CI*：0.077～0.643，*P*＝0.006）可延长OS期（表3）。将上述单因素分析中差异有统计学意义的因素及深部脑组织受累、含HD-MTX化疗为自变量，以患者的OS为因变量，进行多因素Cox回归分析。结果显示：含HD-MTX化疗（*HR*＝0.082，95％*CI*：0.008～0.873，*P*＝0.038）、接受auto-HSCT治疗（*HR*＝0.151，95％*CI*：0.030～0.747，*P*＝0.020）是OS的独立预后因素。接受含HD-MTX化疗、auto-HSCT治疗可延长OS期。上述因素针对PFS进行相关统计分析，组间差异均无统计学意义（均*P*>0.05）。

**表3:** 49例原发中枢神经系统淋巴瘤患者总生存的单因素及多因素预后分析

因素	单因素分析		多因素分析	
	*HR*（95％ *CI*）	*P*值	*HR*（95％ *CI*）	*P*值
年龄>60岁	3.436（1.008～11.710）	0.049		
女性	1.403（0.487～4.042）	0.531		
多病灶	0.905（0.309～2.652）	0.855		
深部脑组织受累	1.662（0.520～5.307）	0.391	1.606（0.353～7.302）	0.540
血清LDH升高	1.183（0.381～3.674）	0.771		
ECOG体能状态评分≥2分	2.017（0.571～7.125）	0.276		
双表达阳性	0.806（0.245～2.657）	0.723		
Ki-67阳性指数≥80％	1.461（0.448～4.768）	0.530		
IELSG预后危险分层高危	0.207（0.027～1.608）	0.132		
MSKCC评分系统分层高危	22.130（4.736～103.400）	<0.001	2.284（0.657～7.941）	0.194
含HD-MTX化疗	0.110（0.003～3.764）	0.221	0.082（0.008～0.873）	0.038
含BTKi治疗	1.171（0.277～4.942）	0.830		
接受auto-HSCT治疗	0.223（0.077～0.643）	0.006	0.151（0.030～0.747）	0.020
接受CAR-T细胞治疗	0.606（0.171～2.149）	0.438		

**注** LDH：乳酸脱氢酶；ECOG：美国东部肿瘤协作组；IELSG：国际结外淋巴瘤协作组；MSKCC：美国纪念斯隆-凯特琳癌症中心；HD-MTX：高剂量甲氨蝶呤；BTKi：布鲁顿酪氨酸激酶抑制剂；auto-HSCT：自体造血干细胞移植；CAR-T细胞：嵌合抗原受体T细胞

## 讨论

PCNSL是一种较为罕见的结外淋巴瘤类型，常见于免疫缺陷特别是HIV感染患者。PCNSL发病率在50～70岁达到峰值，患者的中位年龄为56岁，男女比例为3∶2。据报道，既往20年内，60岁以上老年患者的发病率有所上升[2],[4]。

本研究中位发病年龄为57岁，与文献报道一致[9]，未见明显男女比例差异。PCNSL主要症状和体征因神经系统受累区域而异，但系统性淋巴瘤常见的B症状（发热、盗汗和体质量减轻）在PCNSL中罕见[5]。文献报道脑部受累占30％～50％[15]，主要症状表现为头痛、神经功能缺损症状、神经精神和行为变化、颅内压升高、癫痫发作等。本研究受累部位占比与既往报道一致，并展示了不同临床症状临床发生率情况。约60％的中枢神经系统DLBCL涉及幕上脑组织，较少受影响的部位包括后颅窝（13％）和脊髓（1％）；60％～70％患者存在单一肿瘤，其余病例表现为多灶性疾病[16]。本研究显示大部分患者为多部位受累，常见受累部位为额叶及基底节区，与国内相关报道类似[17]。对于预后评分，IELSG因需要初治时LDH结果，而部分非首诊患者缺乏相关资料。MSKCC评分系统相对简单，仅依靠年龄及KPS评分。本研究显示MSKCC评分系统分级3级为不良预后因素，与文献报道一致[12]。随着对PCNSL机制研究的深入，整合来自多组学数据的全基因组数据揭示了PCNSL中的4种分子模式，具有独特的预后影响[18]，是否需要参考M7-FLIPI评分，将基因结果纳入预后评分体系，仍需进一步研究确认。

目前认为PCNSL存在多种遗传学异常，如9p24.1拷贝数异常或易位、BCL6易位、6p21缺失等，但MYC及BCL2易位少见；多种常见基因突变，包括MYD88、CD79B、CDKN2A、PIM1等，其中MYD88^L265P^突变发生率明显高于系统性DLBCL[7],[10]。Yuan等[19]对68例初治PCNSL患者的组织样本进行全基因组测序，发现IGLL5、PIM1、MYD88、CD79B、BTG、KMT2D、TBL1XR1、PCLO、HIST1H1E和BTG1是较为常见的基因突变，本研究检出的常见基因异常与文献报道一致，但因数量有限，与临床特征及预后未见明确相关性。Liang等[20]研究显示，对于判断中枢神经系统受累情况，脑脊液循环肿瘤DNA（ctDNA）检测较其他传统检测方式具有更高的灵敏度；并构建CNSi-IPI模型预测中枢神经系统受累情况。Wang等[21]研究显示，在85.7％的新诊断PCNSL患者的脑脊液游离DNA（cfDNA）中可以检测到≥1种突变基因，cfDNA持续阳性的患者PD更迅速，其比MRI可更早预测PD。目前基因检测及监测仅用于科研阶段，未进行临床普及，本研究患者治疗后临床常规送检脑脊液ctDNA阳性率极低，考虑与检测技术相关。未来病理组织或脑脊液相关基因检测或可用于指导靶向药物使用及监测或预测病情变化。

对于可耐受化疗的PCNSL患者，其一线化疗方案仍然是包含HD-MTX（≥3.5 g/m^2^）的联合化疗方案。IELSG32研究显示，MATRix方案CR率可达49％、ORR达87％，MATRix方案7年PFS率高达52％、MATRix方案和巩固治疗的患者7年OS率为70％[22]–[23]。但其不良反应发生率较高，尤其是重度骨髓抑制和粒细胞缺乏伴发热发生比例较高。本中心未发表数据显示，MATRix方案治疗耐受性较差，大部分患者需要减量或更换方案。新药时代亟需寻找高效低毒的治疗方案，目前的研究策略是在HD-MTX联合抗CD20单抗基础上，再联合BTKi、IMiD或PD-1抑制剂等新药。本研究亦显示，含HD-MTX的一线治疗方案可达较高缓解率，其中HD-MTX联合新药方案较传统HD-MTX缓解率略高，组间差异虽无统计学意义，且非随机对照研究，但该类方案组合为寻找有效一线治疗方案提供了有益临床数据资料。

临床中存在部分患者因年龄、基础疾病、或存在严重并发症及合并症（如气管插管、重症肺炎等）等情况，不耐受含HD-MTX方案治疗，临床证实选择无化疗药物治疗方案可以使患者疾病得到稳定或缓解，可避免出现化疗药物不良反应，使后续治疗方案的实施及疾病获得治愈成为可能。类似方案不同药物组合在临床广泛探讨[24]。

关于一线治疗后患者的巩固治疗及维持治疗，IELSG32研究显示[25]，全脑放疗（WBRT）和auto-HSCT均为有效巩固治疗手段，可显著提高CR率，中位随访30个月，WBRT后CR率为95％、auto-HSCT后CR率为93％。MATRix方案和auto-HSCT并未导致更高的非复发死亡率或第二肿瘤发病率，接受WBRT治疗的患者的注意力和执行功能受损，而接受auto-HSCT治疗的患者的注意力、执行功能、记忆力和生活质量均有所改善。

本研究中，未进行任何形式巩固维持治疗患者的疾病复发率为60％；接受巩固维持治疗患者的疾病复发率为32％，提示一线治疗后有必要采取巩固维持治疗维持缓解状态。本研究仅1例患者采用放疗进行巩固治疗（多在复发/难治状态采用放疗），无法比较放疗与其他治疗方式的差异。采用auto-HSCT巩固的12例患者仅1例复发，采用药物巩固维持的患者16例患者中8例PD，auto-HSCT存在一定生存优势趋势。在单因素及多因素分析中，接受auto-HSCT治疗患者有较长生存。以上均表明auto-HSCT在巩固治疗中的重要地位。然而在实际临床工作中，存在部分患者无法有效动员出自体造血干细胞，此时需寻找其他可能维持治疗方案。目前临床广泛采用新药作为维持治疗，包括BTKi、IMiD及PD-1抑制剂等药物。auto-HSCT后患者是否需采用维持治疗，目前暂无相关资料。本研究显示，auto-HSCT后采用BTKi维持治疗患者中暂无复发病例，未采用维持治疗患者中1例复发。若患者可耐受BTKi，可考虑接受维持治疗，从而延长PFS及OS期。但auto-HSCT后维持治疗的利弊，仍需随机对照研究进行验证。

综上，本研究提示对于PCNSL患者，高龄及预后危险分层高危患者预后较差，采用含HD-MTX治疗方案，进行auto-HSCT可改善患者生存，未来仍需大规模数据进一步验证。
